# Four Cases of Morbus Coxarius Successfully Managed, and Calculated to Show the Precise Treatment in the Various Stages of That Formidable Disorder

**Published:** 1839-01-19

**Authors:** William Harris


### MEDICAL EXAMINER.

DEVOTED TO MEDICINE, SURGERY, AND THE COLLATERAL SCIENCES.

No. 3.] PHILADELPHIA, SATURDAY, JANUARY 19, 1839. [Vol. II.

*Four Cases of Morbus Coxarius successfully managed, and calculated to show the precise treatment in the various stages of that formidable disorder.* By William Harris, M. D.

*Case First.*

M. J., aged five years, when I was first called to see her, had slight tumefaction of her right hip, with considerable tenderness to the touch, pain in the knee of the affected side, the leg considerably flexed upon the thigh, and the thigh slightly upon the body. Her temperament was leuco-phlegmatic, and her general health tolerably good. When she attempted to walk, she only touched the floor with the toes of the affected limb, and every effort gave her pain in the hip.

It was clearly a case of coxalgia. She was immediately put to bed, and several cups applied around the joint, which took away about four ounces of blood. The next morning a dose of cream of tartar and jalap was administered, which operated well. The tenderness to the touch had somewhat abated, but every effort to extend the limb gave her great pain. She continued in bed, was restricted in her diet, purged every other day, and cupped again on the eighth day. After the second cupping, I applied Dr. Physick's long splint for fracture of the os femoris, and made gentle extension and counter-extension, which gave the little patient some pain, but succeeded, in a measure, in straightening the limb. After four hours it was removed, and the next day renewed, at the same hour, when it was allowed to remain on eight hours. This application gave the child less pain, and straightened the limb more. The splint was renewed daily, extending the time of the application; so that in a week from the commencement, it was allowed to remain on all the time, and at the end of two weeks the limb was nearly straight, and the patient free from suffering. This part of the treatment, I acknowledge, is at variance with professional usage. Its success, however, is the best argument in its favour. The father of my patient being in indigent circumstances, I was unwilling to put him to the expense of purchasing a carved splint, and therefore procured a binder's board, one-fourth of an inch thick, sufficiently long to reach from the middle of the thorax to the ankle, and of appropriate dimensions as to width, which, of course, was greater above than below. On either side of this splint, opposite the acetabulum, I made an incision from without, obliquely inwards and downwards, leaving between the two cuts a space of half an inch in the centre. I now caused the outer edges of the cuts to overlap, and secured them in that condition with a strong ligature. I then caused an older child to go to bed,—and, after soaking my splint, thus prepared, in boiling water, I applied it in its wet and pliable condition to his right limb, in a straight attitude, and, having moulded it carefully, it was secured with a roller, and allowed to remain on twenty-four hours. At the end of this period, I had a hard, dry, and well-moulded splint, which, after lining it with a layer of carded cotton, was applied to my patient, with which she was much pleased, and which made her very comfortable. From this time forward she was purged twice a week with various kinds of purgative mixtures, such as cream of tartar and jalap, rhubarb and magnesia, Seidlitz powder, Epsom salts, &c., and indulged in a pretty generous diet, consisting of mush and milk, bread and milk, with a little poultry or mutton, and rice, at dinner. She was quite comfortable and contented, and enjoyed pretty good health during the whole of her confinement to bed, which was eleven months. At the end of that time the splint was removed, and the patient allowed to move her limb gently, or to suffer another to move it, but was not permitted to get out of bed until the end of the twelfth month. During the last month, she would cry every night at bedtime, unless her mother would re-apply the splint for the night, declaring that she could not sleep without it. How wonderful is the force of habit! She was now mounted upon crutches, the limb being a fourth of an inch shorter than the other. At the end of three months after she arose from bed, she was nearly well, enjoying, in a great measure, all the ordinary powers of her limb. At that time she left the neighbourhood,—but, I believe, she entirely recovered.

*Case Second.*

J. M., of Phœnixville, Chester county, a German mechanic, requested me to visit his daughter, then fourteen years of age, who, he said, had had a running issue in her thigh upwards of nine years, the disease having commenced before he left Germany. Upon entering his humble habitation, I observed in the object of the father's solicitude, a female near the age of puberty, crawling upon the floor, pallid, extremely emaciated, and in a very feeble state of health. She had not attempted to walk for several years, even with the help of crutches; but moved upon her nates, backwards, by the assistance of her hands and arms. Upon examining the affected limb, I found the knee flexed upon the thigh, and the thigh upon the body, but there was slight passive motion in both joints. From the history of the case it was clearly of a coxalgic character; and there was, at this time, a large fistulous opening in the front of her thigh about five inches below Poupart's ligament, which was discharging very copiously a thin unhealthy pus. I could trace the opening to the neighbourhood of the hip-joint, but had no reason to think I penetrated it with the probe, nor did I detect any carious bone. I ordered a compound syrup of sarsaparilla, consisting of sarsaparilla root, the bark of the root of sassafras, and senna, &c., with two grains of corrosive sublimate to the bottle. Of this she took a wine-glass half full three times a day. It acted as an alterative and mild aperient, and at the end of a month her health was decidedly improved. I now commenced injecting daily up the fistulous sinus a streng solution of sulphate of copper, and, after a fortnight, when the thigh had become very sore to the touch throughout the whole course of the sinus, I applied a thick compress in its direction, and passed over it and around the thigh a very tight bandage. The sulphate of copper having eaten away the surface of the fistulous ulcer, new and healthy granulations were shooting forth, which, by means of the compress and roller, were brought in contact, and a complete obliteration of the whole canal was in this way accomplished in a few weeks.

After the discharge had ceased and the ulcer was completely healed, her abdomen began to swell, and I was very apprehensive of abdominal dropsy. I increased her medicine, however, which purged her pretty freely, put her on a rigid diet, and in a fortnight more the swelling subsided. Her health now improved under the use of the syrup—her appetite, her flesh, and her strength increasing pretty rapidly.

The knee and hip-joint, at present, admitted of but slight passive motion; and when she rested upon the tuberosity of the ischium and heel, the ham of the diseased limb could not be pressed nearer than within four inches of the floor. I measured the distance upon a piece of cedar shingle—then reduced its length half an inch, and directed her father, by friction with warm oil and pressure, to endeavour to bring the ham down to the shortened stick against my next visit, which would be at the end of a week. This was accomplished,—and, by a similar course, at the end of eight weeks the knee was straight, and admitted of pretty free motion. The same treatment was, at the same time, adopted, to improve the passive motion of the hip-joint, and with the same success. She, now, supported by crutches, and assisted by her friends, walked for the first time for several years. In six months after, she could walk well, without any assistance,—was free from lameness, and was one of the most florid, fat, and healthy girls I had ever seen. She afterwards married, and, I believe, still continues to enjoy vigorous health.

*Case Third.*

J. D., aged fourteen years, had been labouring under a coxalgic affection for two years, which had advanced to the suppurative stage nearly a year before I was requested to visit him. Upon examination I found that a dislocation of the hip-joint had taken place—that the limb was two inches shorter than the other, and that there were five fistulous openings in the neighbourhood of the joint, which were discharging large quantities of unhealthy pus. Upon injecting warm water into one of the openings, it passed out at the other four, carrying with it large quantities of matter. I next examined with the probe, and found the head of the bone in a carious state. The compound syrup of sarsaparilla was administered, the same as in the preceding case, and with the happiest effect. His health, which, at first, was very feeble, began to improve;—the ulcers were washed twice a day with Castile soap and warm water, and dressed sometimes with bread and milk poultice, and again with simple cerate and patent lint. There was very little improvement in the condition of the ulcers during the first three months of my attendance. The health of the patient, however, was decidedly improved, and his spirits were good. About this time the head of the thigh-bone sloughed off from its carious neck, and came away. I now commenced injecting into the fistulous ulcers the solution of sulphate of copper, and treated the ulcers with simple cerate, compress, and tight roller, in the same manner as in the preceding case, and with the same success. In three months from the time the head of the bone came away, the five fistulous ulcers were all perfectly healed. During the process of healing the ulcers, the neck of the bone was forming a ligamentous attachment to the dorsum ilii. The patient was now, by the aid of crutches, enabled to walk abroad; the new joint gradually grew stronger; and two years afterwards, when I accidentally met him, I found that a strong ligamentous union had formed, which admitted of flexion, extension, and rotation. It was, for all useful purposes, a complete joint—which enabled him, now, to walk all day without the aid of crutches, and with little more than the ordinary fatigue. The limb was two inches shorter,—and, could this have been prevented, he would now have been free from lameness. This desirable end, I think, would have been attained, if, at the time the head of the bone came away, the patient had been placed in Hagedorn's apparatus for fractured thigh, as improved by Professor Gibson, and by extension and counter-extension the limb had been brought down to the proper length, and kept in that situation until ligamentous union had formed between the neck of the bone and some point of the innominatum back of the acetabulum. The health of this patient is now quite robust.

*Case Fourth.*

General George M. Brooke, of the U. S. Army, commanding at Fort Howard, Green Bay, having a son afflicted with coxalgia, which was likely to terminate in permanent deformity and lameness, determined to send him to Philadelphia, that he might receive the best surgical treatment.

Accordingly, Mrs. Brooke, with her son, whose name is William, and a servant, set out from Green Bay, under the care of a friend of the family, in the latter end of May, 1835, and arrived in this city about the last of the following month.

A distinguished surgeon was immediately called in, who, after a thorough examination of the child's hip-joint, pronounced the case incurable. Mrs. Brooke afterwards invited me to see her son. She informed me that the disease had commenced fifteen months before she left Fort Howard; that it was induced by an injury; that at the end of eight months an abscess had formed involving the hip-joint, which broke and discharged for four months; that at the end of this period the abscess healed, and the limb was found to be shorter than the other; and that he had not been able to bear any weight upon that limb since the disease commenced.

The little sufferer was but four years of age, of a sanguine temperament, and of robust health.

Upon examination, I discovered that the right hip-joint was dislocated; that the head of the os femoris was lodged upon the dorsum ilii; that the right limb was about two inches shorter than the other; and that, in moving across the floor, he hopped upon the left leg, the right one hanging uselessly by his side. I gave it as my judgment that he might be cured; but was not willing to undertake the process of restoration which I had in contemplation upon my own responsibility, and requested that a gentleman of the profession in whose surgical skill I had the fullest confidence, should be called in consultation. Upon examination, he agreed with me as to the condition of the joint, but united with the gentleman first consulted in the opinion that the case was incurable. Upon proposing to him, however, a plan of treatment which the result of the last case had suggested, he consented that the experiment should be tried.

The mother eagerly embraced the proposition; and, accordingly, we at once set about making the necessary preparations to carry into effect my plan of treatment, which was to consist of permanent extension and counter-extension. As it had not been more than three or four months since the head of the bone had slipped from its socket, it was not likely that it had formed any strong adhesions in its new location; and, if not, I should be able, by a suitable apparatus, to overcome the contractions of the muscles—to bring down the limb to its proper length, and to keep it there until nature should have time to form a new joint.

I accordingly had made Hagedorn's apparatus for fractured thigh, as improved by Professor Gibson, of appropriate dimensions, and two days afterwards I commenced the treatment. It was now the fourth of July, and the weather very warm. I had the patient placed, therefore, in an airy room, upon a small table previously covered with a mattress and a linen sheet, with a pillow under his head. I had at the same time another table, of the same size, placed in his room, with a circular opening near its centre, about nine inches in diameter, to which was affixed an appropriate tin vessel. This arrangement answered the purpose. As often as he wished *aller à la selle*, it was very easy to slip him from one table to the other without disturbing the apparatus. For a description of the apparatus, I refer the reader to Professor Gibson's Surgery, plate xii., page 294. When I first placed the splints upon the child, and attempted to draw down the foot of the affected limb to the foot-board, the effort gave him considerable pain. The muscles, indeed, were so rigidly contracted, that the first trial was without effect. Besides, our patient's sufferings were so great, that we did not think it prudent, the first day, to keep him in the apparatus more than an hour. At the suggestion of the consulting surgeon, the integuments of the joint were rubbed freely twice a day with an ointment composed of twelve grains of iodine, four scruples of hydriodate of potassa, fifty drops of oleum nicotianæ, and two ounces of adeps præparata, which had a very relaxing effect upon the muscles. The second day our patient bore the extension and counter-extension two hours with less suffering, and with some success. The foot, by this effort, was brought a quarter of an inch nearer the foot-boards, which gave us some encouragement,—although it returned as soon as the extending straps attached to the gaiters were loosed. The splints were re-applied every day, gradually increasing the time of their application and the extending force applied to the affected limb. Three weeks, however, had elapsed before we were enabled to keep the apparatus permanently applied. The little patient was now very comfortable, and, after three weeks more, the foot of the shorter limb was brought in contact with the foot-board. But, as the ends of the bones forming the joints of the sound limb were pressed close together, and as those of the diseased limb were drawn apart as far as the ligaments would allow them, and long-continued extension had probably lengthened them a little, I caused an excavation to be made, half an inch deep, in the foot-board opposite the foot of the diseased side,—and, at the end of two more weeks, this foot was brought in contact with the bottom of the excavation.* I was now satisfied that if nature would form a ligamentous joint, with the limb in this situation, we should have a radical cure, and without deformity. As well as I could ascertain, the head of the bone was now resting against the os innominatum immediately behind the acetabulum. Mrs. Brooke was now highly delighted with the prospect of her son's restoration,—and, feeling confident that she could carry out the plan of treatment at Green Bay, was very anxious to return. And, accordingly, on the 8th of September, she turned her face homewards. Her son, extended in the apparatus, travelled upon his back in steam-boats, canal-boats, lake-boats, and rail-road cars, from this place to Fort Howard, fifteen hundred miles, without once having his feet detached from the foot-board. I shall be mainly indebted to my correspondence with General Brooke and

* This may prove a profitable hint to those who treat oblique fractures of the thigh-bone with this apparatus.—W. H.

others, for the remainder of the narrative of this case.

Extract of a letter from General BROOKE, dated "*Fort Howard*, October 12th, 1835.

"Doctor Macomb, of the Army, happened to pass this way with some recruits, and examined William particularly. His hips and legs are perfectly alike, and the Doctor thinks the head of the bone has returned completely to the socket. When the gaiter is loose from the foot-board, he uses his leg with the greatest natural ease, throwing it up from the splints. The limb has ceased to contract, when loose, for more than two weeks. We shall, however, continue him in the splints, and upon his diet, until all danger has passed, probably at least two months more."

The apparatus was continued, according to my direction, from the commencement until it was finally removed, about one year. During all that period the boy enjoyed excellent health, and was very contented and happy.

Extract of a letter from General BROOKE, dated "*Fort Howard*, November 12th, 1836.

"This will be handed you by my friend, Mr. Benjamin L. Webb, whom I asked to call this morning, for the purpose of looking at William. He will be much more able to describe, orally, the appearance of his leg and hip, than I can by writing. The case, we all think, will be nearly a perfect cure. The lameness is scarcely perceptible, if at all. His leg is, perhaps, the eighth of an inch shorter than the other,—and, by lying on his back, and pressing a little on the knee, there is no difference. Both surgeons Satterlee and Wright, the attending physicians of the garrison, are delighted with the cure, and think the application of the splints in cases of hip-joint disease worthy of all praise."

Mr. Webb, to whom the General refers in his last letter, was disappointed in his intended visit to Philadelphia, but wrote to me a letter dated "New York, January 23d, 1837," of which the following is an extract:

"Under the expectation that I should visit your city on my return from the West, I called, at the solicitation of General Brooke, at Fort Howard, to see a child of his, in order that you might be informed of the cure wrought by means of your treatment of him when painfully afflicted with hip-joint disease. I found his child, apparently about six years of age, capable of walking without the assistance of any crutch at all. He was requested by his mother to "march," and his long strides evinced to me that he experienced no pain by the action. He was then requested to take hold of a crutch, and, leaning upon it, to swing his leg around as high as it could be raised,—and he did so, throwing it forward, backward, and sideward, in the most perfect manner. He then lay down, and his leg was measured, which was about a quarter of an inch shorter than the other. I am, in my own mind, satisfied that, in this instance, a perfect cure is effected. His parents both consider it a remarkable recovery; and the General desired me to say to you, sir, that he should be happy to have you use, in any way, the statement of his son's case, that would promote the interest of the medical profession."

According to the testimony, then, of General Brooke, it appears that "the cure is nearly perfect," "the limb is, perhaps, an eighth of an inch shorter," and if the knee be pressed down, "there is no difference," "the lameness is scarcely perceptible." Mr. Webb, however, testifies that "the limb is one-fourth of an inch shorter than the other," that the joint admitted of extension, flexion, and rotation, that he *marched* with the long, easy strides of a soldier, and he considered that "a perfect cure is effected."—Whether there is a line and a half, or three lines, or "no difference" in the length of the limbs—the fact that he has a good joint, and that he can walk without crutches, and with "lameness scarcely perceptible, if at all," I am free to confess that a more perfect cure has been accomplished, than I ever, in my most sanguine moments, anticipated.

A few months after this, I received a letter from Mrs. Brooke, which has been unfortunately mislaid, and cannot now be found, containing the melancholy intelligence, that William, owing to laying aside his crutches too soon, or to using his limb too freely, or to another injury, had a return of his hip disease. Another abscess, involving the new joint, had broken, and was then discharging, and the limb had again become shorter.

I advised that the original plan of treatment be adopted immediately, which was done, and continued several months.—The same good effect followed.—The abscess soon healed, the limb was again brought down, a ligamentous joint again formed, and the boy is again upon his feet. During his last confinement to the apparatus, the extension, and counter-extension, used, was not sufficiently powerful, as his leg is now precisely half an inch shorter than the other. He is still a little lame, but can walk without crutches, though he is still using them as a matter of prudence. He is in fine health, which, indeed, has never suffered from either of his long confinements. He will soon be able to walk upon his limb all day without crutches, and with but very little lameness. The cure, however, is not as perfect as it was after the first effort.

The history of this case has fully satisfied me that the extending and counter-extending apparatus, timely applied, and continued a sufficiently long time, will prevent any shortening of the limb, or deformity from coxalgia, and I furthermore believe, that if I had kept William Brooke in Philadelphia, under my own care, his restoration would have been complete. Far be it from me to attach blame to any person;—but no one could reasonably expect that another would carry out a new plan of treatment with the same attention and care as the individual with whom it originated.

